# The combination of rapamycin and MAPK inhibitors enhances the growth inhibitory effect on Nara-H cells

**DOI:** 10.3892/ijmm.2014.1715

**Published:** 2014-04-02

**Authors:** OSAMU NAKAMURA, TOSHIAKI HITORA, YOSHIKI YAMAGAMI, MASAKI MORI, HIDEKI NISHIMURA, RYOSUKE HORIE, KONOSUKE YAMAGUCHI, TETSUJI YAMAMOTO

**Affiliations:** Department of Orthopaedic Surgery, Kagawa University School of Medicine, Kagawa 761-0793, Japan

**Keywords:** autophagy, apoptosis, mammalian target of rapamycin, mitogen-activated protein kinase

## Abstract

The inhibition of the mammalian target of rapamycin (mTOR) signaling pathway promotes the initiation of autophagy, and the mitogen-activated protein kinase (MAPK)/extracellular signal-regulated protein kinase (ERK) is well known to induce autophagy. Autophagy is a self-defense mechanism of cancer cells that are subjected to antitumor agents, and blocking autophagy can trigger apoptosis. In the present study, we demonstrate that an mTOR inhibitor, rapamycin, induces autophagy in the Nara-H malignant fibrous histiocytoma (MFH) cell line through the activation of ERK1/2. Rapamycin-induced apoptosis was enhanced following the inhibition of the MEK/ERK pathway. In the Nara-H cells, we examined the effects of rapamycin treatment on cell proliferation and on the phosphorylation of the mTOR pathway components and autophagy by western blot analysis. Furthermore, we examined the effects of rapamycin with or without the MEK inhibitor, U0126, on the induction of apoptosis by using fluorescence microscopy. Rapamycin inhibited Nara-H cell proliferation and decreased the phosphorylation of the mTOR pathway in the Nara-H cells. Rapamycin induced the apoptosis of Nara-H cells, and this apoptosis was enhanced by U0126. Simultaneously, phospho-ERK1/2 was activated by rapamycin. The present study demonstrates that rapamycin induces autophagy in Nara-H cells by activating the MEK/ERK signaling pathway, and the rapamycin-induced apoptosis can be enhanced by the MEK inhibitor, U0126. These results suggest that self-protective mechanisms involving mTOR inhibitors in Nara-H cells are prevented by the inhibition of the MEK/ERK pathway. The combination of an mTOR inhibitor (e.g., rapamycin) and an MEK inhibitor (e.g., U0126) may offer effective treatment for MFH, as this combination effectively activates apoptotic pathways.

## Introduction

The mammalian target of rapamycin (mTOR) is an essential serine/threonine kinase that belongs to the phosphoinositide-3-OH kinase (PI3K)-related kinase family (1). It is an intracellular protein that mediates cell growth, proliferation, migration and survival and promotes angiogenesis in many types of cancer (2,3). The inhibition of mTOR affects pathway-mediated transcription and translation, leading to cell cycle arrest and anti-angiogenesis. As regards the antitumor mechanisms of mTOR inhibitors, certain studies have indicated that inhibitors of Akt and its downstream target, mTOR signaling, have antitumor effects as the inhibition of this pathway contributes to the initiation of autophagy (4–6).

mTOR nucleates two complexes, the mTOR complex 1 (mTORC1) and the mTOR complex 2 (mTORC2). mTORC1 is activated by growth factors, nutrients and the cellular energy status. mTORC1 increases mRNA translation through the activation of ribosomal p70S6 kinase (p70S6K), the inhibition of eIF4E-binding protein (4EPB1) and autophagy [autophagy-related protein (Atg)13].

Autophagy has gained attention due to its paradoxical roles in cell survival and cell death, particularly in the pathogenesis and treatment of cancer (7). The regulation of autophagy is highly complex and includes input from the cellular environment through the PI3K/Akt/mTOR pathway (8). Not surprisingly, there is an intricate relationship between autophagy and apoptosis. Recent studies have indicated that autophagy can function as a self-defense mechanism in cells that are subjected to antitumor agents and that blocking autophagy can trigger the activation of apoptosis (9–11).

Mitogen-activated protein kinase (MAPK)/extracellular signal-regulated protein kinase (ERK) is a key molecule in intracellular signal transducing pathways that transport extracellular stimuli from the cell surface to the nuclei (12). A previous study demonstrated that U0126, a MAPK inhibitor, blocks MAPK/ERK signaling and decreases cell proliferation in osteosarcoma (OS) and malignant fibrous histiocytoma (MFH) (13).

The MAPK and mTOR pathways have multiple cross-connections that allow the coupling of cell-cycle activation to the regulation of protein translation. Rapamycin and its analogs activate the MAPK pathway in human cancer, which indicates that there is a novel mTORC1-MAPK feedback loop (14). On the other hand, other studies have indicated that the inhibition of the mTORC1 signaling pathway promotes the initiation of autophagy (15), and the PI3K/Akt/mTOR and MAPK/ERK pathway are two significant signaling pathways regulating autophagy (16). Therefore, we hypothesized that the combination of rapamycin and MAPK inhibitors may enhance the growth inhibitory effect on tumor cells.

Soft tissue sarcomas account for only 1% of adult malignancies, 60% of which are located in the extremities. MFH is one of the most common soft tissue sarcomas in adults; it was first described in 1963 by Ozzello *et al* (17) and then in 1964 by O’Brien and Stout (18). In recent years, drugs that target specific molecules have been developed as treatments for human malignancies, including these sarcomas (19). These drugs often selectively inhibit specific molecules, such as growth factor receptors or intracellular signaling proteins that are related to tumor proliferation, migration and/or metastasis (20). In this study, we focused on the mTOR and MAPK/ERK signaling pathways.

The aim of the present study was to examine the effects of the mTOR inhibitor, rapamycin, on Nara-H cells (an MFH-derived cell line). We examined whether rapamycin affects the suppression of the phosphorylation of proteins in the mTOR pathway and/or the induction of autophagy though the activation of MAPK/ERK in Nara-H cells. Furthermore, we examined whether the combination of rapamycin and a MAPK inhibitor induces apoptosis in Nara-H cells.

## Materials and methods

### Chemical reagents

Rapamycin (CCI-779) was purchased from Calbiochem (San Diego, CA, USA), dissolved in dimethyl sulfoxide (DMSO) and stored at −20°C. The MEK inhibitor, U0126, was purchased from Promega (Madison, WI, USA), dissolved in DMSO, and stored at room temperature.

### Cell lines and cell culture

The Nara-H cells were purchased from ScienStuff Co. (Nara, Japan). The Nara-H cell line was established from a myxoid MFH of the uterus by Kiyozuka *et al* (21). The cells were grown in Dulbecco’s modified Eagle’s medium (DMEM; Sigma-Aldrich, St. Louis, MO, USA) containing 10% fetal bovine serum (FBS; Sigma-Aldrich) and 100 U/ml penicillin. The cells were routinely maintained at 37°C in a humidified 5% CO_2_ atmosphere, and cultures were used at the mid-log phase.

### In vitro proliferation assay

Cell proliferation was determined by the CellTiter 96^®^ AQueous One Solution Cell Proliferation assay (Promega). The cells were trypsinized and seeded at a density of approximately 1×10^4^ cells/well in 96-well cell culture plates with 200 μl culture medium containing 10% FBS and incubated for 48 h. Following this initial incubation, the growth medium was replaced with medium containing 10% FBS and rapamycin at a concentration of 0, 0.4, 2, 10 or 50 μM. After 24 and 48 h, the medium was removed, the cells were washed with phosphate-buffered saline (PBS), and fresh medium containing 3-(4,5-dimethylthiazol-2-yl)-5-(3-carboxymethoxyphenyl)-2-(4-sulfophenyl)-2H-tetrazolium (MTS) reagent (100 μl medium plus 20 μl MTS regent/well) was added to each well. In the experiments testing the combined effect of rapamycin and U0126, the cells were treated with 40 μM rapamycin and 50 μM U0126 for 24 h. In the experiments testing the effect of rapamycin or U0126, the cells were treated with 40 μM rapamycin or 50 μM U0126 for 24 h. The optical density was measured at 490 nm with an automatic microplate reader after 2 h of further incubation following the addition of the MTS reagent. The absorbency is directly proportional to the number of living cells. The percentage viability of each well was calculated. At least three independent experiments were performed.

### Western blot analysis

The cells were trypsinized and seeded at a density of approximately 6×10^5^ cells/well in 6-well cell culture plates in 2 ml culture medium with 10% FBS. After 48 h, the cells were treated with 10% FBS containing rapamycin at the concentration of 0, 0.4, 2, 10 or 50 μM for 24 h. In the experiments testing the combined effect of rapamycin and U0126, the cells were treated with 40 μM rapamycin and 50 μM U0126 for 24 h. In the experiments testing the effect of rapamycin or U0126, the cells were treated with 40 μM rapamycin or 50 μM U0126 for 24 h. Following treatment, the culture medium was replaced with lysis buffer (Cell Signalling Technology, Beverly, MA, USA), and the cells were lysed on ice for 20 min. The cell lysates were spun at 15,000 × g using the Tabletop Micro Refrigerated Centrifuge 3500 (Kubota Shoji Co. Ltd., Tokyo, Japan) at 4°C for 30 min. The supernatant was then collected as the total cellular protein extract. The protein concentrations were determined using the Protein Assay Bicinchoninate kit (Nacalai Tesque, Inc., Kyoto, Japan) and standardized with bovine serum albumin. The samples of total cellular protein were loaded onto a SDS polyacrylamide gel (10% or 12.5% commercial precast gel; Wako, Tokyo, Japan), and the proteins were separated by SDS-PAGE under reducing conditions. The separated proteins were electrophoretically transferred onto nitrocellulose membranes (GE Healthcare Bio-Sciences, Piscataway, NJ, USA). The membranes were blocked for 90 min in blocking buffer that contained tris-buffered saline (TBS-T) and EzBblock Chemi (Atto Co., Tokyo, Japan). The membranes were then incubated overnight at 4°C with primary antibodies (Table I) that were diluted in the blocking buffer. The specific HRP-conjugated secondary antibody incubations were performed overnight at 4°C with gentle agitation. Bound antibodies were detected by using the ECL plus western blotting detection system (GE Healthcare Bio-Sciences) and the LAS-1000 plus image analyzer (Fujifilm Co., Tokyo, Japan). Specific signals were quantified by densitometric analysis using NIH ImageJ software.

### RNA interference

The cells were trypsinized and seeded at a density of approximately 1×10^6^ cells/well in 6-well cell culture plates in 2 ml culture medium with 10% FBS. After 48 h, the cells were washed with PBS and transfected with mTOR small interfering RNA (siRNA; mTOR siRNA1 was purchased from Cell Signalling Technology) using the X-tremeGENE siRNA Transfection Reagent (Roche Applied Science, Penzberg, Germany) according to the manufacturer’s instructions. Following transfection, the cells were incubated for 48 h before the extraction of total RNA.

### Quantitative reverse transcription-polymerase chain reaction (qRT-PCR)

Total RNA was extracted from the cells using Isogen (Nippon Gene, Tokyo, Japan). The RNA was then reverse-transcribed into cDNA using High Capacity cDNA Reverse Transcription kits (Applied Biosystems, Foster City, CA, USA) for RT-PCR. Quantitative PCR was performed on an Eco™ Real-Time PCR System (Illumina, Inc., San Diego, CA, USA) using Power SYBR^®^-Green PCR Master Mix (Applied Biosystems). The primers (mTOR, Atg5 and Beclin 1) for quantitative PCR were synthesized and validated by Hokkaido System Science Co., Ltd (Hokkaido, Japan). Atg5 is a gene product required for the formation of autophagosomes (22). Beclin 1 is also known as Atg6, which plays a key role in autophagy (23). All primers used in these preparations are listed in Table II.

### Fluorescence microscopy images of cells expressing pEGFP-LC3

The cells were trypsinized and seeded at a density of approximately 1×10^6^ cells/well on 25-mm circular coverslips (Matsunami Glass Ind. Ltd., Osaka, Japan) in 2 ml culture medium with 10% FBS overnight. The cells were then transfected with the pEGFP-LC3 plasmid using X-tremeGENE HP DNA Transfection Reagent (Roche Applied Science) and incubated for 24 h. The pEGFP-LC3 plasmid was purchased from Addgene (Cambridge, MA, USA; Addgene database plasmid 21073), which was provided by Dr Tamotsu Yoshimori (Osaka University, Osaka, Japan). The cells were then washed with PBS and treated with rapamycin and/or U0126 for 24 h. Following treatment, the cells were imaged in an Attofluor cell chamber (Molecular Probes/Invitrogen Life Technologies, Carlsbad, CA, USA) on the thermo-controlled stage (Tokai Hit INU-ONI, Shizuoka, Japan) of an inverted epifluorescence microscope (Carl Zeiss LSM 700 confocal laser scanning microscope; Carl Zeiss Inc., Thornwood, NY, USA). Autophagy was evaluated by examining the punctate forms (type II) of the autophagic marker, LC3, based on pEGFP-LC3. Autophagy was quantitated by the percentage of GFP-LC3-positive autophagic vacuoles or cells with LC3 punctate dots.

### Fluorescence microscopy images of Annexin-V FITC-stained cells

The cells were trypsinized and seeded at a density of approximately 1×10^6^ cells/well on 25-mm circular coverslips in 2 ml culture medium with 10% FBS for 48 h. The cells were then washed with PBS and treated with rapamycin and/or U0126 for 24 h. Following treatment, the cells were incubated with Annexin V-FITC and PI using an Annexin-V-Fluos Staining kit (Roche Applied Science) for 15 min in a dark room. The cells then were imaged in an Attofluor cell chamber on the thermo-controlled stage of an inverted epifluorescence microscope, as described above.

### Electron microscopy

The cells were trypsinized and seeded at a density of approximately 1×10^4^ cells/well on a Lab-Tek^®^ Chamber Slide (Nalge Nunc International, Naperville, IL, USA) in 1 ml culture medium with 10% FBS for 48 h. The cells were then treated with rapamycin and rapamycin plus U0126 for 24 h. For transmission electron microscopy, the treated cells were washed and fixed with 2.5% glutaraldehyde in 0.1 M phosphate buffer (pH 7.4) for 2 h and then post-fixed with 1% osmium tetroxide in the same buffer for 2 h. They were dehydrated in a graded series of ethanol and embedded in Epon 812. Ultrathin sections were stained with 4% uranyl acetate and lead citrate and observed under an electron microscope (JEM-1400; Jeol, Tokyo, Japan) at 80 kV.

### Statistical analysis

Statistical analyses for the cell proliferation assay and quantitative PCR were performed using GraphPad Prism 5 software (GraphPad, San Diego, CA, USA) with one- or two-way ANOVA, followed by post-hoc analysis. A value of p<0.05 was considered to indicate a statistically significant difference.

## Results

### Rapamycin inhibits the proliferation of Nara-H cells

**Figure 1:**
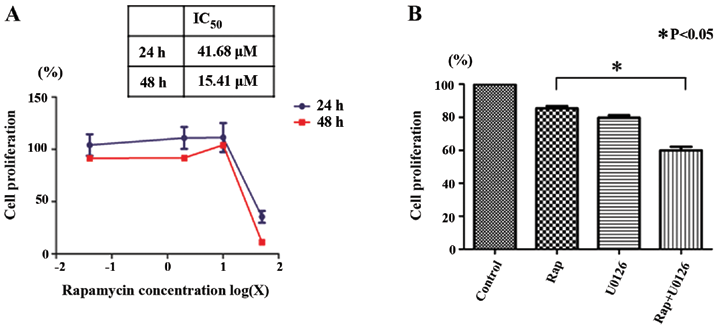
Cell proliferation assay was used to investigate the effects of rapamycin on the proliferation of cultured Nara-H cells. (A) Rapamycin inhibited Nara-H cell proliferation in a dose- and time-dependent manner. (B) Nara-H cell proliferation was lower in the rapamycin plus U0126-treated cells than in the rapamycin-treated cells (p<0.05). Rap, rapamycin.

We examined the effects of rapamycin on Nara-H cell proliferation using the CellTiter 96^®^ AQueous One Solution Cell Proliferation assay to determine whether rapamycin inhibits cell proliferation. Rapamycin inhibited Nara-H cell proliferation in a dose- and time-dependent manner. The IC_50_ for 24 h of rapamycin treatment in the Nara-H cells was 41.68 μM (Fig. 1A).

### Rapamycin-induced Nara-H cell death is enhanced by U0126

We then examined the effects of rapamycin with or without U0126 on Nara-H cell proliferation. Based on the IC_50_ of rapamycin after 24 h, we examined the proliferation of the Nara-H cells treated with 40 μM rapamycin (Rap group), 50 μM U0126 (U0126 group), and 40 μM rapamycin plus 50 μM U0126 (Rap + U0126 group) for 24 h. Cell proliferation was significantly lower in the Rap + U0126 group than in the Rap group (p<0.05) (Fig. 1B). These results indicate that U0126 enhances the rapamycin-induced suppression of Nara-H cell proliferation.

### Western blot analysis

**Figure 2:**
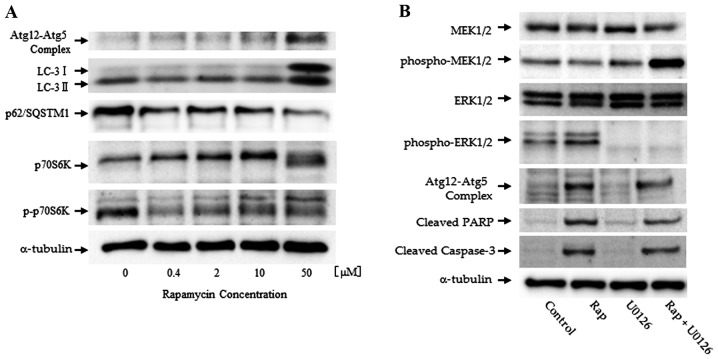
Western blot analysis was used to investigate the effects of rapamycin on components of the mammalian target of rapamycin (mTOR) pathway. (A) Phospho-p70S6 kinase (p70S6K) expression levels were decreased following treatment with rapamycin in a dose-dependent manner. (B) Analysis of the effects of treatment with rapamycin and/or U0126. Treatment with rapamycin resulted in a dose-dependent increase in LC-3II and Atg12-Atg5 complex expression. On the contrary, p62/SQSTM1 expression was decreased in a dose-dependent manner. Cleaved PARP and cleaved caspase-3 expression was increased by rapamycin and rapamaycin-plus-U0126 treatment. Rap, rapamycin.

Western blot analysis demonstrated that treatment with rapamycin induced the phosphorylation of p70S6K, one of the key components in the mTOR pathway. Additionally, we examined the expression of the Atg12-Atg5 autophagy-related gene complex, p62/SQSTM1, and LC-3 in Nara-H cells exposed to various concentrations of rapamycin (ranging from 0.4 to 50 μM) for 24 h (Fig. 2A). Treatment with rapamycin resulted in a dose-dependent decrease in the levels of phospho-p70S6K, which is a downstream effector of mTOR. These findings indicate that rapamycin affects the mTOR pathway by inhibiting the phosphorylation of downstream effectors of mTOR. LC-3II and Atg12-Atg5 complex expression was used as an autophagic marker. p62/SQSTM1 is a polyubiquitin-binding protein, which is degraded by autophagy (24). Treatment with rapamycin resulted in a dose-dependent increase in the expression of LC-3II and the Atg12-Atg5 complex in the Nara-H cells. On the contrary, p62/SQSTM1 expression was decreased in a dose-dependent manner (Fig. 2A).

Subsequently, the MAPK signaling pathway and the expression of autophagic markers were evaluated. LC-3II expression was increased by treatment with rapamycin. Simultaneously, the phosphorylation of ERK1/2, which is one of the downstream effectors of MAPK/ERK, was increased by rapamycin. In the cells treated with rapamycin and rapamycin plus U0126, cleaved PARP and cleaved caspase-3 were strongly expressed (Fig. 2B).

### Quantitative PCR

**Figure 3:**
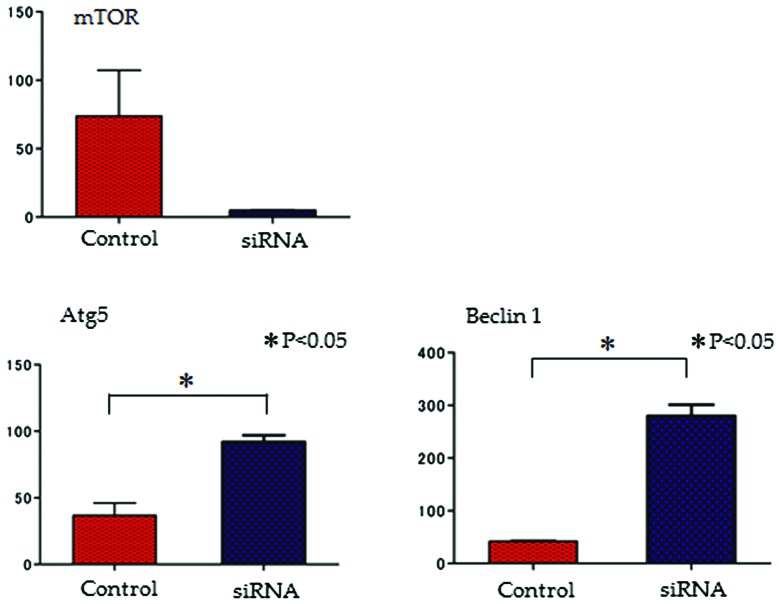
mRNA expression of mammalian target of rapamycin (mTOR) and the autophagy-related genes, Atg5 and Beclin 1, was evaluated following transfection with mTOR siRNA. mRNA expression of mTOR was decreased and autophagy-related gene expression was increased by mTOR siRNA.

To determine the effects of the knockdown of the mTOR signaling pathway, the mRNA expression of mTOR, Atg5 and Beclin 1 was evaluated following transfection with mTOR siRNA. The expression of both autophagy-related genes (Atg5 and Beclin 1) was increased by mTOR siRNA; on the contrary, the mRNA expression of mTOR was decreased (Fig. 3).

### Fluorescence microscopy images

**Figure 4:**
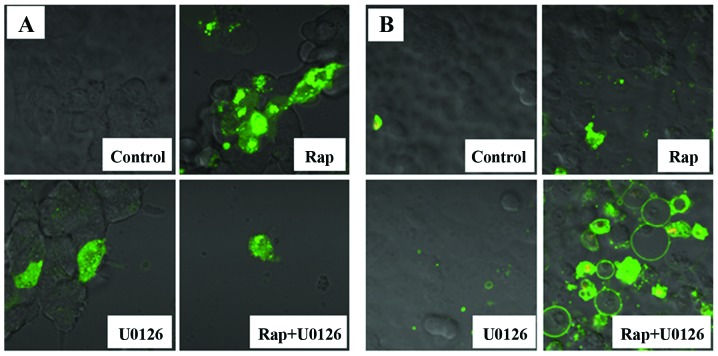
Fluorescence microscopy images. (A) The expression of pEGFP-LC3 was used to evaluate autophagy. pEGFP-LC3 dot formation was significantly increased by rapamycin treatment. (B) Apoptotic cells were detected by Annexin V and PI staining. There were more Annexin V-stained cells in the group treated with both rapamycin and U0126 than in the other groups. Rap, rapamycin.

The expression of pEGFP-LC3 by fluorescence microscopy, in which green fluorescent protein (GFP) is expressed as a fusion protein at the amino terminus of LC3, was used to evaluate autophagy. pEGFP-LC3 dot formation was markedly increased in the Rap group (Fig. 4A).

We then used Annexin V-FITC and PI to detect the apoptotic cells. Annexin V-FITC is a marker for early apoptosis, and PI is a marker for late apoptosis and necrosis. We observed several Annexin V-FITC-positive cells (early stage of apoptosis) and high number of Annexin V-FITC plus PI-positive cells (late stage of apoptosis) in the Rap + U0126 group (Fig. 4B). The number of apoptotic cells was greatly increased in the Rap + U0126 group as compared to the controls, the U0126 group or the Rap group.

### Electron microscopy

**Figure 5:**
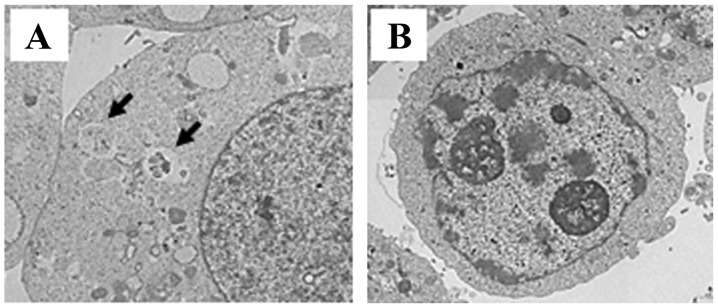
Electron microscopy. (A) Arrows indicate autophagosomes detected following treatment wiht rapamycin. (B) Nuclear fragmentation and chromatin condensation in the nucleus were detected in the rapamycin plus U0126-treated group.

Electron microscopy revealed that the autophagosomes were detected on the rapamycin-treated cells (Fig. 5A). Nuclear fragmentation and chromatin condensation in the nucleus were detected in the Rap + U0126 grou (Fig. 5B). Chromatin condensation and oligonucleosomal DNA fragmentation are the nuclear hallmarks of apoptosis (25).

## Discussion

### Soft tissue sarcomas

Soft tissue sarcomas, particularly high-grade sarcomas, such as MFH, are clinically aggressive and frequently metastasize to various organs. In the absence of effective systemic chemotherapeutic treatments for aggressive sarcomas, targeted therapies are being investigated and used as treatments. Targeted drugs, including mTOR inhibitors, are currently being tested as single agents or in combination with other agents, such as autophagic inhibitors, for the treatment of sarcomas (26–28).

### mTOR pathway

A variety of cell signaling events in the PI3K/Akt pathway are mediated by mTOR, and this pathway plays a central role in cell survival and proliferation in many types of cancer (27). mTOR exerts its biological functions as being part of two different protein complexes, mTORC1 and mTORC2. mTORC1 regulates autophagy, consisting of the mTOR catalytic subunit, regulatory associated protein of mTOR (raptor), G protein β-subunit-like protein (GβL, also known as mLST8) and proline-rich Akt substrate of 40 kDa (PRAS40). mTORC2 consists of mTOR, rapamycin-insensitive companion of mTOR (rictor), GβL, SAPK-interacting protein 1 (SIN1) and protein observed with rictor (PROTOR) and is not a direct regulator of autophagy (29,30). Rapamycin, a specific inhibitor of mTORC1, has been shown to selectively and completely block the mTORC1-dependent p70S6K phosphorylation and partially block 4EBP1 phosphorylation (31).

### mTOR inhibition and induction of autophagy

As previously demonstrated, the inhibition of the Akt/mTOR pathway is consistently associated with triggering autophagy in cancer cells (32). Recent studies have also indicated that autophagy can function as a self-defense mechanism in cells that are subjected to antitumor agents and that blocking autophagy can trigger the activation of apoptosis (9–11). Based on these findings, it has been suggested that inhibitors of autophagy, such as 3-methyladenine (3-MA), may be an effective treatment for MFH as they activate apoptosis. In our previous study, we demonstrated that the combination of an mTOR inhibitor and an autophagic inhibitor (temsirolimus and 3-MA, respectively) was an effective treatment for Nara-H cells as this combination effectively activated the apoptotic pathways (27). In the present study, our results revealed that rapamycin suppressed the phosphorylation of p70S6K; on the other hand, rapamycin increased the expression of the Atg12-Atg5 complex and LC-3II. These results indicate that suppression of the mTOR pathway is involved in the induction of autophagy.

### Rapamycin-induced autophagy and activation of MAPK pathway

As a critical signaling pathway involved in tumorigenesis, the MAPK/ERK signaling pathway is often activated in numerous types of cancer cells. Accumulating evidence indicates that the activation of ERK1/2 is associated with autophagy (33). Saini *et al* reported that there is a significant level of cross-talk between the mTOR and MAPK/ERK pathways and that the inhibition of one cascade activates the other (34). Thus, the inhibition of the Raf/MEK/ERK pathway leads to increased activity in the PI3K/AKT/mTOR pathway, possibly through the loss of feedback inhibition. Similarly, blocking the PI3K/AKT/mTOR pathway leads to increased activity in the MAPK/ERK pathway (34). In the present study, we examined the expression and constitutive phosphorylation of molecules involved in the MAPK/ERK signaling pathway in Nara-H cells. We found that rapamycin indeed activated the ERK1/2 and Atg12-Atg5 complex. These results indicate that rapamycin induces autophagy and activates the MAPK/ERK signaling pathway.

### mTOR inhibitor and MAPK inhibitor

The present study demonstrates that apoptotic cell death and strong anticancer efficacy result from the use of MAPK inhibitors, inhibiting autophagy. Apoptotic cell death in the Nara-H cells was illustrated by the expression of cleaved caspase-3 and PARP by western blot analysis (Fig. 2B) and by fluorescence microscopy (Fig. 4B) and electron microscopy (Fig. 5). The inhibition of MEK1/2 by U0126 inhibited the expression of the Atg12-Atg5 complex, thus enhancing the cytotoxic effects of rapamycin. The inhibition of mTORC1 or mTORC2 by the transient or moderate activation of MEK/ERK moderately enhanced Beclin 1 expression, resulting in cytoprotective autophagy, whereas the inhibition of both mTORC1 and mTORC2 by sustained MEK/ERK activation strongly induced Beclin 1 expression, leading to cytodestructive autophagy (35). Indeed, Beclin 1 gene expression in the Nara-H cells was increased follwoing the knockdown of mTOR by siRNA (Fig. 3B). Therefore, it is thought that the combination of mTOR inhibitors and MAPK inhibitors may result in strong antitumor effects (14).

In conclusion, the present study demonstrates that rapamycin induces cytoprotective autophagy in Nara-H cells by activating the MEK/ERK signaling pathway and that rapamycin-induced apoptosis can be enhanced by MEK inhibitors. These results suggest that self-protective mechanisms involving mTOR inhibitors in Nara-H cells are prevented by the inhibition of the MEK/ERK pathway. In other words, the MEK/ERK signaling pathway is activated by rapamycin as a self-defense mechanism, and cytoprotective autophagy is induced. The combination of an mTOR inhibitor (e.g., rapamycin) and an MEK inhibitor (e.g., U0126) may offer effective treatment for MFH, as this combination effectively activates apoptotic pathways.

## Figures and Tables

**Table I:** Primary antibodies used in western blot analysis.

Target	Source	Host	Dilution	Secondary antibody	Gel (%)
MEK1/2	Cell Signaling	Rabbit	1:1,000	Anti-rabbit	10
Phospho-MEK1/2	Cell Signaling	Rabbit	1:1,000	Anti-rabbit	10
p44/42 MAPK (ERK1/2)	Cell Signaling	Rabbit	1:1,000	Anti-rabbit	10
Phospho-ERK1/2	Chemicon	Rabbit	1:1,000	Anti-rabbit	10
p70S6	Cell Signaling	Rabbit	1:1,000	Anti-rabbit	10
Phospho-p70S6	Cell Signaling	Rabbit	1:1,000	Anti-rabbit	10
4EBP1	Cell Signaling	Rabbit	1:1,000	Anti-rabbit	10
Phospho-4EBP1	Cell Signaling	Rabbit	1:1,000	Anti-rabbit	10
Cleaved-PARP	BD Biosciences	Mouse	1:1,000	Anti-mouse	10
Cleaved-caspase-3	Cell Signaling	Rabbit	1:1,000	Anti-rabbit	12.5
Atg5-Atg12 complex	BML, Inc.	Mouse	1:1,000	Anti-mouse	10
p62/SQSTM1	BML, Inc.	Rabbit	1:1,000	Anti-rabbit	10
LC-3	BML, Inc.	Rabbit	1:1,000	Anti-rabbit	12.5
α-tubulin	Sigma	Mouse	1:1,000	Anti-mouse	10

**Table II:** Gene-specific primers used for RT-PCR.

Primer	Nucleotide (5′→3′)
mTOR forward	GGA GCT CCA GCA CTA TGT CA
mTOR reverse	TTT CCT CTC ATT GGC ATC TG
Atg5 forward	GCT TGG AGT AGG TTT GGC TT
Atg5 reverse	CAA GTT GGA ATT CGT CCA AA
Beclin 1 forward	CTC TGG CCA ATA AGA TGG GT
Beclin 1 reverse	CGG CAG CTC CTT AGA TTT GT
